# Steam sauna and mother roasting in Lao PDR: practices and chemical constituents of essential oils of plant species used in postpartum recovery

**DOI:** 10.1186/1472-6882-11-128

**Published:** 2011-12-15

**Authors:** Hugo J de Boer, Vichith Lamxay, Lars Björk

**Affiliations:** 1 Department of Systematic Biology, Uppsala University, Norbyvägen 18D, SE-75236 Uppsala, Sweden; 2 Department of Biology, National University of Laos, Dongdok Campus, Vientiane, Lao PDR

## Abstract

**Background:**

Fundamental in traditional postpartum recovery in Lao PDR is the use of hotbeds, mother roasting, steam sauna and steam baths. During these treatments medicinal plants play a crucial role, but little has been published about how the treatments are carried out precisely, which species are used, the medicinal properties of these species, and the medicinal efficacy of their chemical constituents.

**Methods:**

Sixty-five interviews, in 15 rural villages, with women of 4 different ethnic groups were conducted to survey confinement rituals, and postpartum plant use and salience. Essential oils from the main species used were extracted using steam distillation and the main chemical constituents characterized using gas chromatography-mass spectrometry (GC-MS).

**Results:**

A total of 10 different species were used by three or more of the ethnic groups included in this study. All species were used in steam sauna and bath, but only 3 species were used in hotbed and mother roasting. Essential oils of *Amomum villosum, Amomum microcarpum *and *Blumea balsamifera *were found to contain significant amounts of the following terpenes: β-pinene, camphor, bornyl acetate, borneol, linalool, D-limonene, fenchone, terpinen-4-ol and α-terpinene.

**Conclusions:**

Many of these terpenes have documented antimicrobial and analgesic properties, and some have also synergistic interactions with other terpenes. The mode of application in hotbed and mother roasting differs from the documented mechanisms of action of these terpenes. Plants in these two practices are likely to serve mainly hygienic purposes, by segregating the mother from infection sources such as beds, mats, stools, cloth and towels. Steam sauna medicinal plant use through inhalation of essential oils vapors can possibly have medicinal efficacy, but is unlikely to alleviate the ailments commonly encountered during postpartum convalescence. Steam sauna medicinal plant use through dermal condensation of essential oils, and steam bath cleansing of the perineal area is possibly a pragmatic use of the reported medicinal plants, as terpene constituents have documented antimicrobial, analgesic and anti-inflammatory properties.

## 1. Background

The postpartum period is important in many Southeast Asian cultures, and is seen as a time of recovery and often entails a period of confinement ranging from 10 up to 45 days. In accordance with humoral medicine, pregnancy is seen as a hot state; with parturition heat is lost and the woman comes into a state of excess cold, and during the postpartum period care should be taken to restore the mother to equilibrium [1]. Confinement as a treatment involves staying inside and near heat, washing only with hot water, drinking hot drinks, eating hot food, and staying away from drafts [1]. Confinement as a term is fairly broad and can include steam sauna and bathing, mother roasting and hotbeds, dietary proscriptions and consumption of medicinal plant decoctions.

Pregnancy, parturition and the postpartum period are not without risk to the mother and infant. According to the latest data, the infant mortality rate (deaths per 1000 live births) and maternal mortality (maternal deaths per 100 000 live births) for Laos is 60.3 and 660, respectively [2,3]. By comparison, those numbers for Sweden are 3.2 and 3 [2,3]. Maternal mortality occurs mainly during the first week postpartum, and the main causes in East Asia and the Pacific area are in order of mortality: haemorrhage, sepsis/infections, obstructed labor and hypertensive diseases [4].

Steam sauna and bath is common throughout Southeast Asia (for review see [1]), and often involve making a decoction of medicinal plants. The participant may either take a bath directly as is common for the Yao in Laos, Thailand and Yunnan province, China [5-7], sit in a special room into which steam from the decoction is led [1,8], or sit in a tent-like construction with [9,10], or on a seat-less chair over [11], a pot containing the steaming decoction. By lifting the lid of the pot the steam is let out, along with the essential oils and other volatile substances, for inhalation and absorption through the skin, and as the water cools off the mother uses the decoction to cleanse her body.

Mother roasting is culturally related to steam baths, but differs in that the convalescent individual lies on a bed placed above a brazier with charcoal embers on which aromatic plants are laid, thus enabling the essential oils to vaporize. Mother roasting is often done to 'dry out', cease expulsion of lochia, restore the uterus to its pre-pregnancy condition and to alleviate postpartum abdominal pain [1]. The hotbed is also an opportunity to rest, to stay away from daily chores and to strengthen social relations [12]. Mother roasting seems to be confined to Southeast Asia, where it is widespread and reported from Borneo [13], Laos [10,14,15], Malaysia [16], Myanmar [17], the Philippines [18], Thailand [19] and Vietnam [20].

In Laos, postpartum practices and food restrictions are common, and a recent study reports that of women in the capital Vientiane, 93% reported adopting a restricted diet after delivery ('*phit kam*'), and 97% lay on hot beds of embers ('*yu phai*') for a period of an average of two weeks [12]. The hotbed is believed to help to dry up and heal the vagina and uterus, and to relax muscles and decrease pain, and noncompliance is regarded as having negative consequences on both maternal and child health [21]. Modern healthcare associates the hotbed with causing maternal and infant death, poor lactation, and diarrhea [12].

Mother roasting and steam baths involve the use of medicinal plants [5,7,9-11]. In mother roasting ('*yang phai*') and hot bed therapy ('*yu phai*' or '*yu kam*') the plant material is placed on the bed, or directly on the charcoal brazier; in steam baths the plant material is decocted, and the water is used for bathing or applied to the skin for cleansing; and in steam saunas the plant material is placed in hot or boiling water, and the convalescent is placed in a confined area saturated with steam, and subsequently washes herself with the decoction. In all procedures the convalescent is exposed to volatile oils from the plant material through inhalation and topical application. Specific plant species used medicinally in traditional steam saunas and baths have been reported [7,10,11,22], whereas reports on plant species used in mother roasting are few [10,19].

Essential oils are used widely to prevent and treat human disease [23]. Antimicrobial efficacy of essential oils from many plant species against a whole range of organisms has been reported [23,24]; and inhalation of essential oils or their individual volatile terpenes is known to have a significant role in controlling the central nervous system [23].

This study aims to 1) survey and identify the most salient plant species used in postpartum mother roasting and steam sauna in Laos; 2) analyze the main constituents of the essential oils of these species using GC-MS; and 3) evaluate the traditional use of these species in postpartum recovery based on their chemical constituents.

## 2. Methods

### 2.1 Interviews

Semi-structured interviews were conducted from 2005 to 2010 in 15 villages in Nakai District, Khammouane Province, and at the steam saunas at Wat Nakhoun Noy monastery, Naxay Thong District; Vientiane Prefecture and Wat Sop Pa Luang, Vientiane city; Vientiane Prefecture, Lao People's Democratic Republic. A total of more than 65 interviews was conducted with people belonging to 4 ethnic groups: the Lao, Saek, Brou, and Kry Phong. Village group interviews, which were culturally most acceptable, were carried out by female interviewers with female informants, and included the village midwife and mothers (women with 1 or more children). Individual interviews between female informants and interviewers were carried out at the two monasteries in Vientiane Prefecture. The traditions and rituals for delivery, mother roasting, steam sauna and bathing were documented and triangulated through multiple interviews and directed questions concerning details, construction, species, temperatures, volumes, times and periods. The interviews used free listing of medicinal plants per usage (mother roasting, steam sauna, bathing) to elicit information. All interviews were conducted in the homestead; and at the end an informal open-ended interview was conducted to collect individual information of each informant. In general the interviews lasted around 3-4 hours, depending on the informants' knowledge as well as the number of assisting interpreters. During the interviews ethno-botanical information was gathered about the plants' local names, uses, preparations, properties, dosages and availability.

Research permission for this study was granted by the National University of Laos (NUoL), within the bilateral research collaboration framework between Sida-SAREC and NUoL, regulated additionally through a Memorandum of Understanding between Uppsala University (UU) and NUoL. Biological material was transferred between NUoL and Uppsala University following an Access and Benefit Sharing and Material Transfer Agreement signed between UU and NUoL. Prior Informed Consent was obtained from informants involved in interviews and plant collection walks. Ethical approval was not required for this study as research participants were only interviewed on knowledge of plant species used for medicinal purposes. Participants were not subjected to any treatment, were not part of any clinical trials, and no information is presented here that could identify individual participants.

### 2.2 Plant material

Following the interviews, plant material for herbarium vouchers was collected in the surrounding forest with some of the informants. All herbarium vouchers were deposited and identified at the herbarium of the National University of Laos; duplicates were deposited at the herbaria UPS and E. The following herbarium vouchers were associated with the plant material used for GC-MS analysis: VL2112, *Amomum villosum *Lour., Vientiane province, Vangvieng district, Ban Houay Mor Neua, N 18° 48' 28.6', E 102° 31' 36.7'', alt 291 m. Vichith Lamxay et al. 17 Jan. 2010; VL2079, *Amomum villosum *Lour., Champasak province, Paksong district, Bane Kong Ta Youn, N 15° 25' 55.2", E 106° 22' 38.5', alt 1003 m., Vichith Lamxay et al. 26 Aug. 2009. VL 2059, *Amomum microcarpum *C.F.Liang & D.Fang, Oudomxay province, Na Mor district, Ban Nam Pheng, N 21° 00' 38.1'', E 101° 39' 50.8'', alt. 737 m., Vichith Lamxay et al. 19 Aug. 2009.

### 2.3 Essential oils

Fresh plant material was collected in the field together with herbarium vouchers, and the essential oils were extracted *in situ *using steam distillation. Fresh plant material was placed with 2 liters distilled water in a 5 liter round-bottomed flask, connected to a steam distillation column, and heated using a heating mantle or open charcoal fire. Local tap water was used for the condenser, and was left to run, or circulated using a 12 V pump in case no running water was available. The essential oils were collected and stored in sealable 2 ml labeled vials.

### 2.4 Chemical analysis

GC-MS analysis was performed at the Sarawak Biodiversity Centre, Kuching, Sarawak, Malaysia. Essential oils were analyzed using GC-MS to identify their components. GC-MS was performed using an Agilent 6890 N gas chromatograph, directly coupled to an Agilent 5975B mass selective detector (MSD) (Agilent Inc., Little Falls, USA). The column used was an HP-5MS 5% Phenyl Methyl Siloxane capillary column (30 m × 0.25 mm × 0.25 μm) (Agilent Inc., Little Falls, U.S.). GC-MS operating conditions were as follows: injector temperature, 250°C; transfer line, 240°C; oven temperature, increasing from 60 to 240°C at 3°C/min; carrier gas, helium at constant flow of 1.2 ml/min at 250°C; sample injection volume, 1 μl; headspace inlet glass liner, 4.0 mm i.d; splitless purge flow to split vent, 60 ml/min at 0.75 min. MS acquisition parameters were as follows: full scan with scan range 40-400 amu; solvent delay: 5 min. MSD ChemStation E.02.01.1177 (Agilent Inc., Little Falls, U.S.) data acquisition software was used to compare mass spectra of chromatographic peaks with entries of Nist 08 and Wiley 8n databases.

## 3. Results

### 3.1 Postpartum medicinal plant use and GC-MS analysis of essential oil constituents

The survey of postpartum recovery customs and plant use conducted 67 interviews at 17 locations in Khammouane province and Vientiane prefecture, Lao People's Democratic Republic. The 10 species most commonly mentioned (as used by three or more ethnic groups) are reported in Table 1 including usage information on plant part, method and medicinal purpose. Most important of all plant species was the use of *Amomum *spp. for the steam sauna and bath, and *Blumea balsamifera *for mother roasting, hotbed, steam sauna and bath, and their use was reported in every single interview. *B. balsamifera *was available at all locations, but either *Amomum microcarpum *or *A. villosum *was used depending on local availability. Informants in Nakai district reported that *A. microcarpum *could be substituted for *A. uliginosum *in case of local scarcity. Gas chromatography coupled mass spectrometry (GC-MS) of essential oils of *Amomum microcarpum*, and *A. villosum *revealed that both species are rich in volatile terpenoids (Table 2). Essential oil data for *B. balsamifera *was collected from existing literature (Table 3).

**Table 1:** Medicinal plants used mother roasting and steamsauna

Scientific name	Lao name	Vouchers	Part used	Preparation	Medicinal use	Salience
*Blumea balsamifera *(L.) DC. (Asteraceae)	Nad	VL1751; VL 1824; Kool 614	Leaves; Twigs	Steamsauna; Bath; Roasting	Postpartum recovery: Anaemia (dizziness, headache); Puerperal fever; Lactagogue; Postpartum physical recovery; Postpartum secondary haemorrhage; Perineal healing; Retraction of the uterus; Miscarriage recovery	4

*Amomum *spp. (*A. villosum *Lour.* and *A. microcarpum *C.F.Liang & D.Fang**) (Zingiberaceae)	Mak Naeng	VL 2054*; VL 2064*; VL 2079*; VL 2112*; VL 2055**; VL 2059**; VL 2076**	Leaves; Pseudostems	Steamsauna; Bath	Postpartum recovery: Anaemia (dizziness, headache); Puerperal fever; Postpartum secondary haemorrhage; Perineal healing; Retraction of the uterus; Lactagogue	4

*Citrus grandis *(L.) Osbeck (syn. *C. maxima *(Burm.f.) Merr.) (Rutaceae)	Mak Phouk	VL 1753; VL 1397	Leaves	Steamsauna; Bath	Postpartum recovery: Anaemia (dizziness, headache); Puerperal fever; Lactagogue; Postpartum secondary haemorrhage; Perineal healing; Retraction of the uterus	4

*Cymbopogon citratus *(DC.) Stapf (Poaceae)	SiKhay	VL 1813	Whole plants	Steamsauna; Bath; Roasting	Postpartum recovery: Anaemia (dizziness, headache); Puerperal fever; Lactagogue	4

*Alpinia galanga *(L.) Willd. (Zingiberaceae)	Kha	VL1748; VL 1383; VL 1776; VL 1854	Leaves; Pseudostems	Steamsauna; Bath	Postpartum recovery: Anaemia (dizziness, headache); Puerperal fevers; Lactagogue	4

*Artocarpus heterophyllus *Lam. (Moraceae)	Mak Mi	VL 2125; Kool 472	Leaves	Steamsauna; Bath	Postpartum recovery: Anaemia (dizziness, headache); Puerperal fever	3

*Gigantochloa parvifolia *(Brandis ex Gamble) T.Q. Nguyen (Poaceae)	Mai Sod	VL 1833; VL 1742; Kool 471	Leaves	Steamsauna; Bath	Postpartum recovery: Anaemia (dizziness, headache); Puerperal fever; Lactagogue; Postpartum mother recovery; Postpartum secondary haemorrhage, Perineal healing; Retraction of the uterus	3

*Gonocaryum lobbianum *(Miers) Kurz (Icacinaceae)	Kanleuang	VL 1396; VL 1424; VL 1516; Kool 470	Twigs; Leaves	Steamsauna; Bath; Roasting	Postpartum recovery: Anaemia (dizziness, headache); Puerperal fever	3

*Liquidambar formosana *Hance (Altingiaceae)	Somphay	VL 1355; VL 1554; VL 1808	Inner bark	Steamsauna; Bath	Postpartum recovery: Anaemia (dizziness, headache); Puerperal fever	3

*Phoebe lanceolata *Nees (Lauraceae)	Phayven	VL 1353; VL 1511; Kool 538; Kool 619	Leaves; Twigs	Steamsauna; Bath	Postpartum recovery: Anaemia (dizziness, headache); Puerperal fever; Postpartum secondary haemorrhage, Perineal healing; Retraction of the uterus	3

**Table 2:** Major compounds in essential oils from fresh *Amomum *species

		VL2112 *A. villosum*		VL2079 *A. villosum*		VL2059 *A. microcarpum*	
		**Leaves (g)**	**Rhizome (g)**	**Leaves (g)**	**Fruit (g)**	**Leaves (g)**	**Fruit (g)**

Chemical name	CAS number	1000	780	1000	360	700	350

b-pinene	127-91-3	6.3	46.2	19.7	15.2	8.0	4.8

b-phellandrene	555-10-2	6.8	-	-	-	-	-

D-limonene	5989-27-5	8.4	-	8.1	1.2	4.5	0.4

L-fenchone	1195-79-5	8.9	4.8	2.7	2.6	-	-

pino-camphone	547-60-4	5.0	-	-	-	-	-

camphor	76-22-2	-	-	23.2	31.4	-	-

terpinen-4-ol	562-74-3	7.3	8.6	3.6	3.1	-	-

a-terpinene	99-86-5	16.1	1.4	-	-	-	-

a-terpineol	98-55-5	4.5	1.1	-	-	-	-

iso-borneol	124-76-5	3.1	-	-	-	-	-

linalool	78-70-6	-	-	-	-	40.4	25.4

borneol	5655-61-8	-	-	1.8	2.0	6.5	-

nerolidol	7212-44-4	-	-	-	-	22.3	49.4

bornylacetate	76-49-3	-	-	14.5	17.0	-	-

Fresh material oil content (‰)		1.20	0.40	0.20	1.50	0.70	1.40

**Table 3:** Reported compounds in essential oils from *Blumea balsamifera *(L.) DC. leaves

Chemical name	CAS number	**Guenther, 1952 **[65]	**Roi, 1955 **[66]	**Van Duong, 1993 **[67]	**Truyen & Chau, 1999 **[25]	**Bhuiyan et al., 2009 **[68]
α-pinene	7785-70-8					0.48

β-pinene	127-91-3					1.2

D-limonene	5989-27-5		+	+	+	0.2

camphor	76-22-2	75.0	+	+	+	0.1

linalool	78-70-6					1.3

1,8-cineol	470-82-6		+	+	+	

borneol	5655-61-8	20.0	+	25.0	+	33.2

caryophyllene	87-44-5					8.2

ledol	577-27-5					7.1

phytol	7541-49-3					4.6

caryophyllene oxide	1139-30-6					4.1

myrcene	84776-26-1			+		

β-camphene	5794-03-6			+		

β-eudesmol	473-15-4			+		

γ-eudesmol	1209-71-8					3.2

guaiol	489-86-1					3.4

thujopsene	470-40-6					4.4

Fresh material oil content (%)		n.s.	n.s.	0.50	0.2-1.88	0.40

### 3.2 Confinement rituals of postpartum recovery ('yu phai' and 'yu kham')

All informants reported postpartum recovery customs and rituals. The default place for postpartum recovery is the hotbed, where mother and infant rest during most of the day and night. The hotbed is only vacated for the following cleansing routine, which is undertaken 3 times per day: steam sauna, bath, roasting on stool or standing over fire, roasting on hotbed, followed by a return to the normal hotbed temperature. The remaining time is spent on the hotbed, and it is here that the mother eats, nurses and sleeps.

#### 3.2.1 Hotbed ('nang kham' and 'nang phai')

The hotbed ritual commences when the mother goes into labor, and the parturient is guided to lie down on a bamboo bed in the kitchen area of the house that is covered by a pandan mat and fresh leaves of *B. balsamifera*. The bed has been prepared in advance, and either the midwife or a female close relative will have lit a charcoal fire underneath the bed. The charcoal fire is made either on an unbaked clay slab, or in a cooking brazier, and is centered directly under the pelvis. The fire is continually tended to keep both room and bed warm (> 50°C). After delivery the infant is washed, and both mother and infant retreat to the bed. The hotbed period lasts 14-21 days, after which the mother resumes her normal daily routine.

Hotbed is considered an essential part of postpartum recovery, and all informants strictly observed a hotbed period of at least 14 days. The medicinal qualities attributed to the hotbed practice were physical recovery, protection against puerperal fever, alleviating symptoms of postpartum anaemia (dizziness and headaches specifically), stimulating expulsion of the lochia, ceasing postpartum bleeding, perineal healing and as lactagogue.

#### 3.2.2 Steam sauna ('hom') and bath ('ab')

In the steam sauna ritual the mother goes to the washing room (*'san'*), which in most rural Lao raised houses consists of a small annex to the kitchen lacking walls and a roof. Here she sits down on a small stool covered in fresh *B. balsamifera *leaves. In front of her are two aluminum pots, one filled with a decoction of medicinal plants and another with cold river or tap water. The core medicinal plants added to the decoction (Table 1), are added in amounts of 0.3-1.0 kg each to the pot, and heated on a brazier to 95-100°, and subsequently transferred to the washing room. The mother undresses, and covers herself and the pots with a thick blanket, creating a little tent. Inside she lifts the lid of the pot with the hot decoction to let out steam, and stoops to inhale the hot aromatic air. The mother underneath the blanket can be quantified as a cone of approximately 175 liters volume with the mother, the stool, and the pots taking up roughly 75 liters. The remaining 100 liters of air space is available for saturation with evaporated volatile oils. Infusion in boiling water of a volume of < 3 kilos of fresh plant material, could lead to evaporation of 4-20 ml of essential oils (Table 2 and Table 3). Fresh plant material is added to the water when the essential oils have evaporated. The resulting concentrations of evaporated essential oils are possibly high enough to induce physiological effects, either through inhalation or dermal condensation on infected or inflamed perineal areas.

After 20-30 minutes, the mother opens the other pot and mixes the cold water with the hot medicinal decoction to create a mixture at 50-60°C. She then proceeds to cleanse her entire body by splashing hot water from a dipper over her body and washing herself. The cleansing takes 10-15 minutes, and when done the mother wraps herself in a sarong without drying, and goes into the house for mother roasting.

#### 3.2.3 Mother roasting ('yang phai')

Mother roasting, the practice where the mother exposes herself to high temperatures (80-100°C) over hot charcoal embers is done in three different ways: while seated on a chair, while standing crouched over the fire, and while reclining on the bed. In all cases the fire is usually cooled down prior to roasting by putting coarse salt on the embers. The first two practices are interchangeable and done immediately after steam sauna and bathing, and always followed by mother roasting on the bed.

In mother roasting on a chair the mother is seated on an open rattan stool covered in fresh *B. balsamifera *leaves, and a brazier with hot embers is placed underneath at a distance of 20-30 cm from the perineum. In mother roasting over the fire the mother stands spread and slightly crouched over the fire exposing the vagina. In both practices the mother is wrapped in a thick cloth, and remains over the brazier for 10-30 minutes. The purpose of the treatments is reportedly to dry the body and specifically the vagina after the bath, and prevent contamination from the use of towels.

Mother roasting on the bed is similar to the standard hotbed procedure. In this case, however, the fire under the bed is used differently. Instead of being used to keep the mother warm while lying with her infant on the bed (hotbed), in the case of mother roasting it is stoked hot for a medicinal treatment lasting 20-30 minutes, during which the infant is passed to a family member. During mother roasting fresh leaves of *Blumea balsamifera *(L.) DC., *Cymbopogon citratus *(DC.) Stapf, and/or *Gonocaryum lobbianum *(Miers) Kurz are placed on the bed and the convalescent mother lies on them covered by a cotton blanket. *C. citratus *and *G. lobbianum *are used seldom as substitutes of *B. balsamifera *in case of unavailability. Volatile oils are vaporized from the leaves by the heat of the fire and confined underneath the blanket, thus surrounding the mother lying on the bed. The mother inhales essential oil vapors and her skin is both in direct contact with fresh plant material and intensively exposed to the vapors confined under the blanket.

## 4. Discussion

### 4.1 Blumea balsamifera

*B. balsamifera *is rich in essential oils with an oil content of 0.2-1.88% of fresh weight [25]. Here it is reported in postpartum recovery to treat anaemia related afflictions such as dizziness and headache. In addition it is used to treat puerperal fever, serve as a lactagogue, alleviate postpartum secondary haemorrhage, promote perineal healing and retraction of the uterus. It is also used to aid perineal healing and retraction of the uterus during miscarriage recovery. In previous studies it is reported in the treatment of leucorrhea, migraine [26]; for conditions after childbirth [27]; and for postpartum recovery [10].

Its main constituents are borneol and camphor (Table 3), both of which show strong in vitro antibacterial activity [28]. In addition, mixtures of camphor and borneol isolated from other species show inhibitory effects towards several microorganisms, possibly resulting from synergistic interactions [29,30]. Camphor is used in Chinese and Japanese traditional medicine as analgesic and is very common in balms and liniments for external use.

Puerperal fever resulting from microbial infections is frequent, and maternal and neonatal death from *Streptococcus *infections is known to occur [10]. *B. balsamifera *is used during all stages of the hotbed and steam sauna procedure as a way of reducing microbial infections. The use of *B. balsamifera *in the steam sauna, bath and hotbed procedure seems highly relevant for antimicrobial and hygienic purposes.

Its traditional use reported here also involves pain relief, and borneol has demonstrated analgesic effects related to a positive modulating effect of GABA receptors [31]. Analgesic effects of camphor could be due to camphor-induced desensitization of TRPV1 receptors as well as the blocking of TRPA1 receptors [32].

In addition to antimicrobial and analgesic properties, borneol has documented anticoagulant properties [33], which in turn could be a risk factor in traditional use of *B. balsamifera *to stop postpartum bleeding. Excess bleeding is not uncommon following childbirth in rural areas in Laos [10]. Some reservations about the possible analgesic and anticoagulant effect concern the uncertainty on actual uptake in this form of treatment. However receptor-activated responses can be effective also at low doses.

### 4.2 Amomum species

All *Amomum *species are rich in essential oils, and many are used in traditional medicine in Southeast Asia for a range of ailments. Informants in this study reported the use of *Amomum *spp. in postpartum recovery to treat anaemia related afflictions such as dizziness and headache. In addition it is used to treat puerperal fever, serve as a lactagogue, alleviate postpartum secondary haemorrhage, promote perineal healing and retraction of the uterus. In previous studies *Amomum *spp. are reported as emmenagogue; to treat uterine tumors; to ease childbirth, and to promote postpartum recovery [34]. Bonifacy [35] indicates that *A. villosum *is a traditional medicine for treating troubles in pregnancy and various abdominal ills. In Cambodia, a decoction of the rhizome of *A. villosum *is considered to improve the blood circulation, and is used as a tonic given to women after childbirth [36]. *A. microcarpum *is used by the Brou, Saek and Kry in postpartum recovery [10], but no other use is reported in scientific literature. In this study Brou, Saek and Kry Phong informants reported the postpartum use of *A. microcarpum *specifically, and indicated that the leaves and/or fruit are used in steam sauna and baths.

#### 4.2.1 Amomum villosum

The analyzed samples of *A. villosum *show a great variation in terpene spectrum with β-pinene, camphor, bornyl acetate, terpinen-4-ol, fenchone, D-limonene, α-terpinene as the major compounds, but with substantial variation per voucher and plant part (Table 2).

β-pinene has antimicrobial activity, which is possibly accentuated through synergistic effects with other terpene constituents like 1,8-cineol [37], borneol or linalool. Minor components like β-pinene and for the whole plant oil terpineol might support an antimicrobial effect [38].

Camphor is used in Chinese and Japanese traditional medicine as analgesic and is very common in balm and liniments for external use. Camphor shows strong in vitro antibacterial activity [28], and mixtures of camphor and borneol isolated from other species show inhibitory effects towards several microorganisms, possibly resulting from synergistic interactions [29,30]. Analgesic effects of camphor could be due to camphor-induced desensitization of TRPV1 receptors as well as the blocking of TRPA1 receptors [32].

Research on bornyl acetate from *A. villosum *in mice has shown it to have analgesic effects [39], and inhalation has sedative effects [40]. Other interesting data show that bornyl acetate has an antiabortive effect on pregnant mice through modulation of the immunological balance at the maternal-fetal interface [41]. However, in the studied ethnic groups steam sauna is used first after birth, and whether inhalation or transdermal uptake of bornyl acetate has any beneficial effects to the convalescing mother remains unclear.

Terpinen-4-ol is diuretic [42], which corroborates the traditional use of *A. villosum *seeds during pregnancy dysuria in Vietnam [25]. In addition, Terpinen-4-ol shows strong in-vivo inhibitory effects on growth of *Candida albicans *in rat vaginal candadiasis [43]. The doses studied are however higher than could be expected in the steam sauna treatment. Nevertheless synergistic effects could strengthen the activity. Terpinen-4-ol has also been shown to have anti-inflammatory effects by suppressing production of inflammatory mediator factors TNFalpha, IL-1beta, IL-8, IL-10 and PGE2 [44]. In addition, terpinen-4-ol has also been shown to have in vitro antiviral activity [45]. Terpinen-4-ol and α-terpinene are the main constituents of the essential oil of *Melaleuca alternifolia *(Maiden & Betche) Cheel, which has antimicrobial and anti-inflammatory properties supported by a wealth of in vitro data [46], and some clinical data [47]. α-Terpinene in addition is widespread in plant essential oils, and has good antioxidative properties [48].

Fenchone has acute local analgesic effects [49] in an in vitro frog model. Fenchone is also effective as pesticide [50-52] through inhibition of acetylcholinesterase activity [53].

D-limonene exhibits antimicrobial activity like many other terpenes and is a key ingredient in many antimicrobial essential oils [54]. The antimicrobial effect is due to a terpene-induced disturbance of microbial membranes [55]. D-limonene has low human toxicity, and in humans it has been used in humans to treat heartburn due to its gastric acid neutralizing effect; and has confirmed chemopreventive activity against breast and colorectal cancer [56].

#### 4.2.2 Amomum microcarpum

The main components in the essential oil of *A. microcarpum *are linalool, nerolidol and β-pinene, with the leaves containing significant amounts of borneol and D-limonene. The latter and β-pinene have been discussed above.

Several linalool accumulating species are used in traditional medicine. Linalool has a dose-dependent sedative effect on the central nervous system, and hypnotic, anticonvulsant and hypothermic properties are also reported [57]. Park et al. [58] report an in vitro anti-fungal effect. Linalool has also been shown to have an antinociceptive effect, resulting in possible pain reduction [59]. Linalool has acute local analgesic effects from low doses [49] in an in vitro frog model.

Nerolidol arrests and or inhibits growth of parasites such as *Plasmodium falciparum *[60], *Babesia *spp. [61] and *Leishmania *spp. [62] Nerolidol has also been demonstrated to sensitize *Staphylococcus aureus *and *Escherichia coli *to antibiotics [63]. During the steam bath this sensitization could result in synergistic effects with antimicrobial compounds such as camphor, bornyl acetate, D-limonene, terpinen-4-ol and α-terpinene.

## 5. Conclusions

### 5.1 Hotbed and mother roasting

*B. balsamifera *is the main species used in hotbed and mother roasting, whereas the other two reported species, *C. citratus *and *G. lobbianum *have only minor roles as substitutes. During hotbed and mother roasting the mother is lying in constant direct skin contact with leaves of *B. balsamifera*, which are heated from below at 50-70°C for hotbed, and 90-100°C for mother roasting. Evaporation and subsequent inhalation of volatile oils is especially intensive in mother roasting: not only are the leaves strongly heated from below, but also the mother is completely covered by a blanket. Although evaporation and inhalation of volatile oils during mother roasting may be effective, it is not likely to prevent the ailments ascribed to benefit from mother roasting. The main functional role of covering the bed in *B. balsamifera *is likely to be sanitary, since maintaining a high standard of hygiene in the homestead and hotbed is difficult. Covering the bed in fresh leaves exhibiting strong antimicrobial activity [28-30] is an effective way of overcoming this challenge. Hygiene is augmented by frequent steam saunas and baths (2-6 times per day), and refreshing the leaves on the bed prior to each mother roasting.

### 5.2 Steam sauna and bath

Evaporation of 4-20 ml of essential oils of *B. balsamifera *and *Amomum *spp. during the steam sauna procedure would result in high concentrations of components like camphor, bornyl acetate, borneol, D-limonene, linalool, nerolidol, fenchone, terpinen-4-ol and α-terpinene. Although vapors of linalool, terpinen-4-ol and α-terpinene have been shown to have antimicrobial effects on airborne microbes [38], inhalation is not likely to have a significant role in alleviating the ailments reported by the convalescing mothers.

During the steam bath following the steam sauna the mother cleanses the perineum with the medicinal plant infusion. This cleansing is done with the hot water used in the preceding steam sauna. The water cools off during the treatment, rendering the temperature too low for the volatile terpenoids to evaporate. Consequently it is rich in volatile oils and other plant secondary metabolites. A double-blind controlled study on the effect of postpartum episiotomy healing using sitz baths with lavender essential oil found that inflammation and pain relief were significantly improved compared to the control group using povidone-iodine sitz baths [64]. The three-times daily dermal application of the traditional medicinal plant boiled extract, rich in essential oils with documented antimicrobial activity, as is customary in postpartum recovery in Laos, is likely to improve hygiene, clean wounds and sores, and aid wound healing. Synergistic interactions between nerolidol and present terpenes, like camphor, bornyl acetate, D-limonene, terpinen-4-ol and α-terpinene could enhance efficacy of these antimicrobial compounds in inhibition of pathogen growth. Pain relief through analgesic action of camphor, bornyl acetate, fenchone and linalool could alleviate discomfort from parturition wounds.

## Competing interests

The authors declare that they have no competing interests.

## Authors' contributions

HdB, VL, and LB conceived the research. VL was responsible for field research and interviews. LB performed the chemical analysis. VL identified the herbarium vouchers; HdB, VL, and LB processed the data. HdB drafted the manuscript. All authors have read and approved the final manuscript.

## Pre-publication history

The pre-publication history for this paper can be accessed here:

http://www.biomedcentral.com/1472-6882/11/128/prepub
